# Influence of synthesis parameters on crystallization behavior and ionic conductivity of the Li_4_PS_4_I solid electrolyte

**DOI:** 10.1038/s41598-021-93539-4

**Published:** 2021-07-07

**Authors:** Florian Strauss, Jing Lin, Jürgen Janek, Torsten Brezesinski

**Affiliations:** 1 Battery and Electrochemistry Laboratory, Institute of Nanotechnology, Karlsruhe Institute of Technology (KIT), Hermann-von-Helmholtz-Platz 1, 76344 Eggenstein-Leopoldshafen, Germany; 2 Institute of Physical Chemistry & Center for Materials Science, Justus-Liebig-University Giessen, Heinrich-Buff-Ring 17, 35392 Giessen, Germany

**Keywords:** Batteries, Structural materials, Solid-state chemistry

## Abstract

Superionic solid electrolytes are key to the development of advanced solid-state Li batteries. In recent years, various materials have been discovered, with ionic conductivities approaching or even exceeding those of carbonate-based liquid electrolytes used in high-performance Li-ion batteries. Among the different classes of inorganic solid electrolytes under study, lithium thiophosphates are one of the most promising due to their high Li-ion conductivity at room temperature and mechanical softness. Here, we report about the effect of synthesis parameters on the crystallization behavior and charge-transport properties of Li_4_PS_4_I. We show that thermally induced crystallization of Li_4_PS_4_I (*P*4/*nmm*), starting from the glassy phase 1.5Li_2_S–0.5P_2_S_5_–LiI, adversely affects the material’s conductivity. However, both conductivity and crystallization temperature can be significantly increased by applying pressure during the preparation.

## Introduction

Lithium-ion batteries (LIBs) using organic liquid electrolytes represent an indispensable energy-storage technology by powering portable electronics and electrifying transportation. After nearly three decades of continuous developments towards increasing the energy and power density, state-of-the-art LIBs are about to approach their physiochemical limits. However, the demand for energy-dense storage devices is growing, mainly driven by the automotive industry to enable long-range electric vehicles. Replacing the liquid electrolyte with a solid electrolyte (SE) is regarded as a promising route for achieving storage cells with superior energy density by implementation of a lithium-metal anode^1,2^. Various SE materials have been reported in the past showing ionic conductivities comparable to those of liquid electrolytes (≥ 1 mS cm^–1^)^3–6^. Among them, lithium thiophosphates further exhibit favorable mechanical properties, allowing for intimate contact with the redox-active material(s), and could potentially be prepared by scalable wet-chemical methods^3,4,7,8^. Unfortunately, these advantages are accompanied by the drawback of poor (electro-)chemical stability (along with chemo-mechanical degradation), which negatively affects the cycling performance of solid-state battery (SSB) cells^9–15^. To prevent direct contact with the SE, protective coatings on electrode materials and surface modifications (interface engineering) have been investigated^16–19^. Since, in reality, such coatings are never perfectly uniform, SE decomposition still occurs to some extent, being detrimental especially to the long-term battery operation^20–22^.

**Figure 1:**
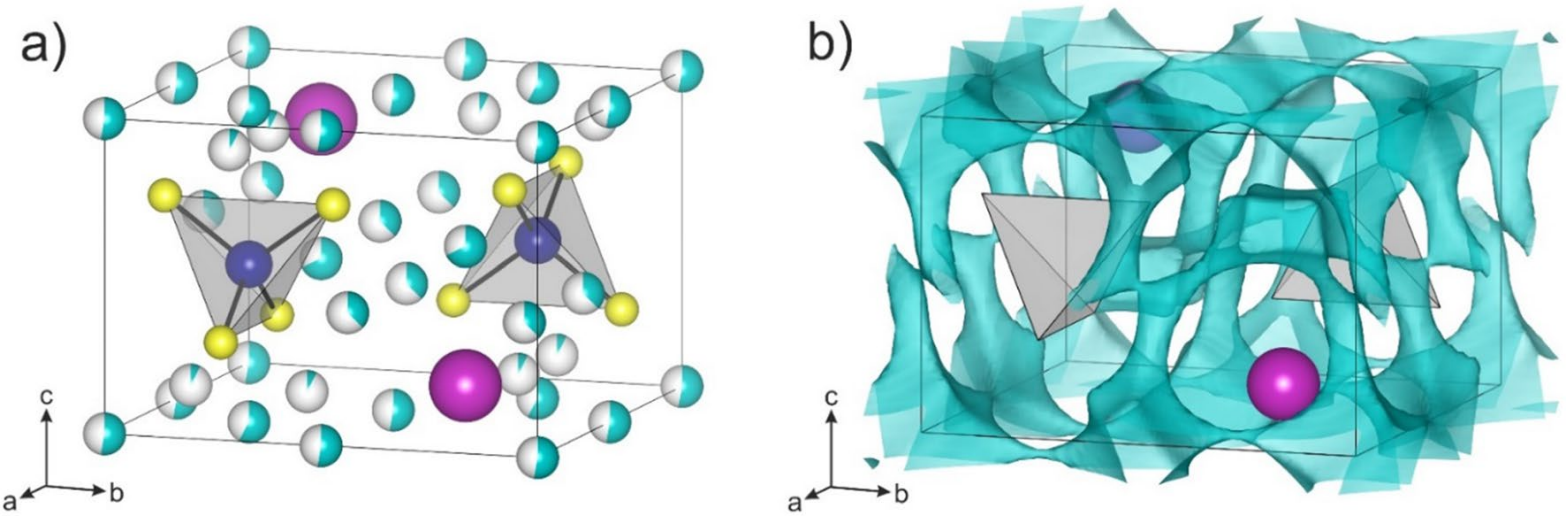
(**a**) Structure of Li_4_PS_4_I crystallizing in the *P*4/*nmm* space group. Li, P, S and I atoms are shown as cyan, blue, yellow and purple spheres, respectively. (**b**) Bond-valence energy landscape showing the 3-dimensional lithium diffusion pathways. For clarity, Li, P and S atoms are omitted. PS_4_^3−^ tetrahedra and I atoms are shown in gray and as purple spheres, respectively.

In recent years, the quasi-ternary *x*Li_2_S–*y*P_2_S_5_–*z*LiI system has attracted much attention, among others, due to the stability of the respective SEs (formation of robust interphases when in contact with cathode or anode materials)^23–28^. An example is the tetragonal Li_4_PS_4_I, which has been identified as a potential new SE^29^. And yet, apparently it only shows a moderate room-temperature (experimental) ionic conductivity in the range between 10^–5^ and 10^–4^ S cm^–1^, regardless of whether prepared by a solution-based method or solid-state synthesis^25,29^. This result is somewhat surprising, as the reported lattice structure contains five partially occupied Li sites situated within the voids between the PS_4_^3−^ tetrahedra and iodide ions, thus offering an open 3-dimensional percolation network for the lithium ions (Fig. 1a,b).

In contrast to experimental observations, recent density-functional theory (DFT) calculations suggest an exceptionally high ionic conductivity of ~ 10^–1^ S cm^–1^ at room temperature^30^. Inspired by this result, in the present work, we re-investigated the 1.5Li_2_S–0.5P_2_S_5_–LiI system and show that crystallization of Li_4_PS_4_I leads to an order of magnitude decrease in conductivity relative to the amorphous counterpart^25^. Specifically, we detail how the synthesis route, temperature and pressure affect both crystallization and conductivity.

The Li_4_PS_4_I SE was prepared at temperatures from 175 to 250 °C using wet-chemical, solid-state and hot-press routes. Structural changes were followed ex situ by X-ray diffraction (XRD). The crystallization was probed using a specialized setup capable of monitoring in situ pressure, temperature and electrical resistance^31^. We demonstrate that the conductivity depends on the synthesis route used and the annealing temperature. Such findings are rationalized with respect to the phase composition and density. Our results also confirm that structural disorder is key to achieving high ionic conductivities in the 1.5Li_2_S–0.5P_2_S_5_–LiI system. Moreover, we show that the applied pressure during heating increases the activation energy for nucleation, thereby increasing the crystallization temperature of Li_4_PS_4_I.

## Results

**Figure 2:**
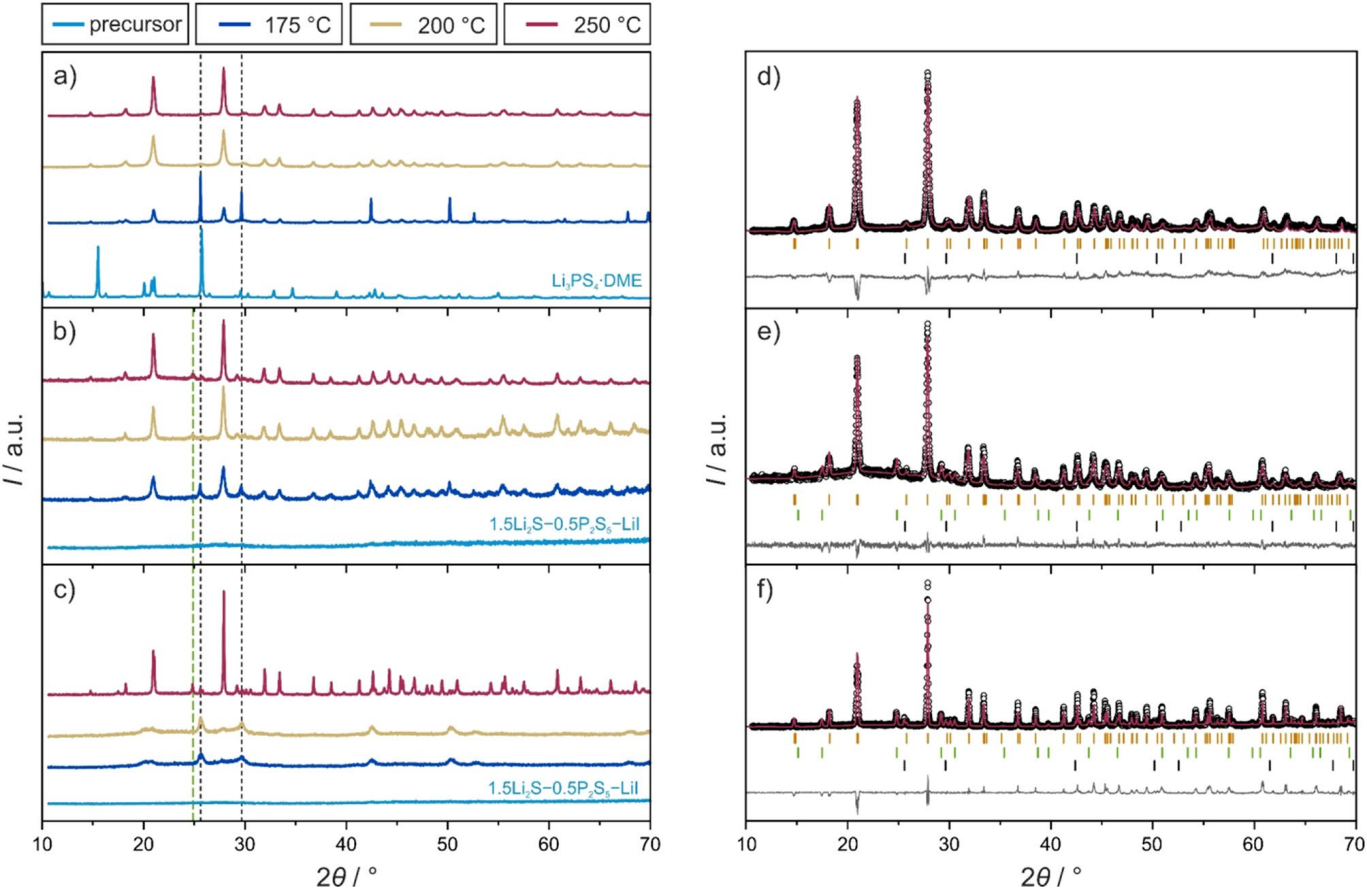
XRD patterns of Li_4_PS_4_I prepared by (**a**) wet-chemical, (**b**) solid-state or (**c**) hot-press synthesis at different temperatures of 175, 200 and 250 °C. Patterns of the corresponding precursors are also shown. Note that for the wet-chemical synthesis, only the Li_3_PS_4_∙DME precursor is shown for clarity, and 1.5Li_2_S–0.5P_2_S_5_–LiI represents the amorphous Li_4_PS_4_I phase produced by ball milling. Green and black dashed lines indicate the main reflections of argyrodite Li_6_PS_5_I and LiI, respectively. Rietveld refinement plots for the samples prepared at 250 °C by (**d**) wet-chemical, (**e**) solid-state or (**f**) hot-press synthesis. Experimental, calculated and difference profiles are shown as black circles and red and gray lines, respectively. Orange, black and green tick marks denote the Bragg reflections of Li_4_PS_4_I (*P*4/*nmm*), LiI (*Fm* − 3*m*) and Li_6_PS_5_I (*F* − 43*m*), respectively. Reliability factors are provided in Table S1.

First, the Li_4_PS_4_I SE was synthesized using a solvent-based approach reported previously^29^. The recovered material from the reaction of preformed Li_3_PS_4_∙DME (1,2-dimethoxyethane) with LiI in DME was annealed at 175, 200 or 250 °C in a vacuum. As shown in Fig. 2a, sharp reflections of unreacted LiI (*Fm* − 3*m*) were present at 175 °C, in addition to minor broad reflections corresponding to Li_4_PS_4_I (*P*4/*nmm*). The reflections of LiI largely vanished upon increasing the temperature to 200 or 250 °C, while those of Li_4_PS_4_I increased in intensity and decreased in broadening. According to Rietveld analysis, the LiI content amounted to ~ 0.2 wt.% after 250 °C (Fig. 2d).

Secondly, the Li_4_PS_4_I SE was prepared by conventional solid-state synthesis. To this end, a mixture of Li_2_S, P_2_S_5_ and LiI was subjected to ball milling to achieve complete amorphization (i.e., 1.5Li_2_S–0.5P_2_S_5_–LiI, see featureless XRD pattern in Fig. 2b). This material was then pressed into pellets and annealed in vacuum-sealed quartz ampoules. Broad reflections corresponding to Li_4_PS_4_I and LiI as major and minor phases, respectively, were visible for the sample at 175 °C. On increasing the annealing temperature, the reflections of Li_4_PS_4_I became even more apparent and the intensity of the LiI reflections decreased. However, small reflections characteristics of argyrodite Li_6_PS_5_I (*F* − 43* m*) appeared. Rietveld refinement resulted in fractions of LiI and Li_6_PS_5_I impurities of ~ 0.4 and 14.1 wt.%, respectively, after 250 °C (Fig. 2e).

Thirdly, the glassy 1.5Li_2_S–0.5P_2_S_5_–LiI as precursor was loaded into a hot press, with the pressure set to 176 MPa (2 t), and heated to the different target temperatures. Surprisingly, at 175 and 200 °C, virtually no crystallization of Li_4_PS_4_I occurred (Fig. 2c). However, broad reflections of LiI were visible. After annealing at 250 °C, pronounced reflections of Li_4_PS_4_I appeared, along with LiI and Li_6_PS_5_I as minor (~ 1.6 wt.%) and major (~ 13.4 wt.%) impurity phases, respectively (Fig. 2f).

Overall, in contrast to the hot-press synthesis, for which the reflections of Li_4_PS_4_I became clearly apparent only at 250 °C, similar crystallization behaviors of Li_4_PS_4_I, with onset temperatures below 175 °C, were observed by XRD for the solvent-based and solid-state synthesis routes.

**Figure 3:**
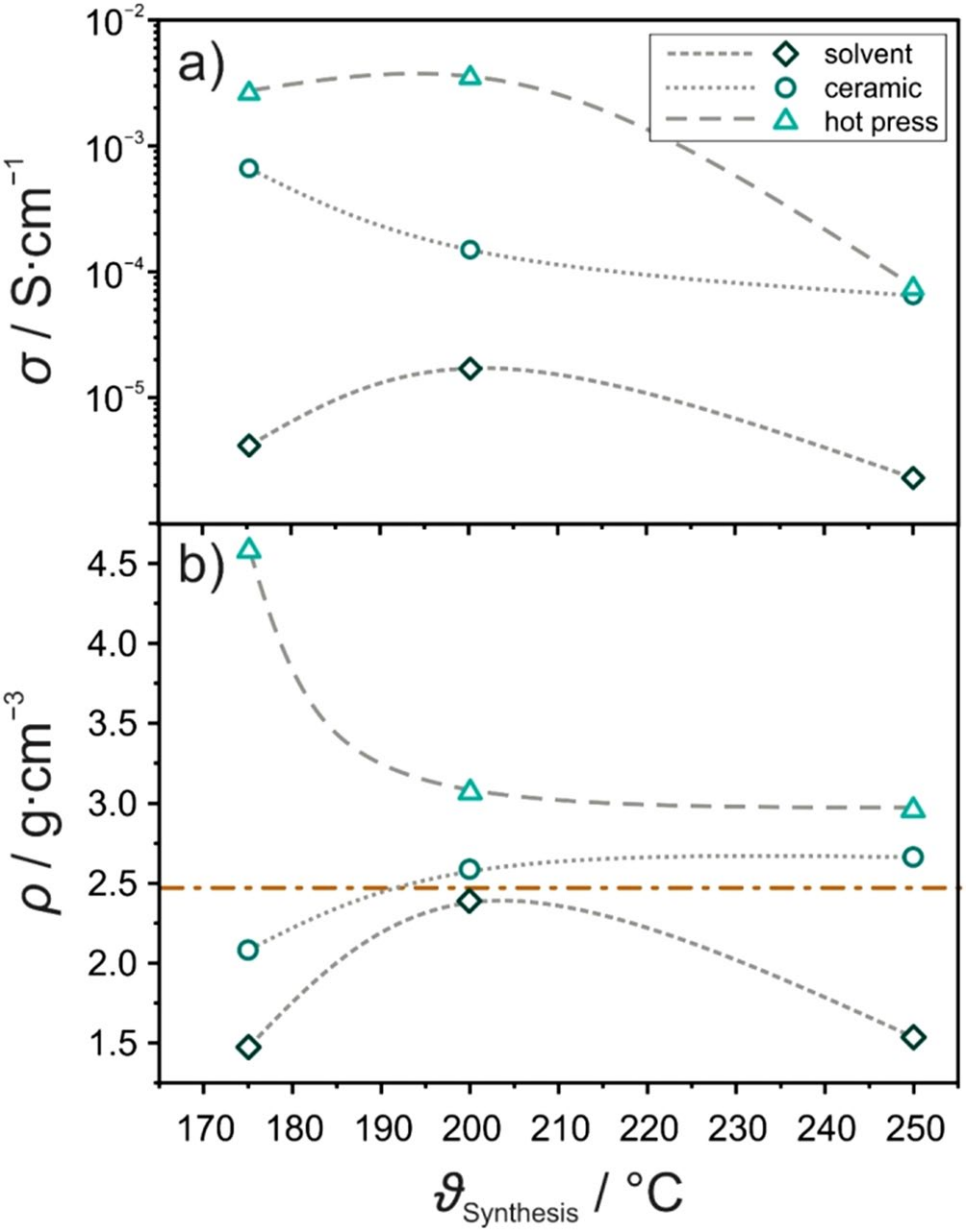
(**a**) Room-temperature conductivities of Li_4_PS_4_I prepared by wet-chemical, solid-state or hot-press synthesis at different temperatures of 175, 200 and 250 °C and (**b**) corresponding experimental densities. Note that the gray dotted/dashed lines are for eye guidance only. The brown line indicates the crystallographic density of tetragonal Li_4_PS_4_I.

The changes in conductivity with structural evolution for the different synthesis routes were followed by conducting electrochemical impedance spectroscopy (EIS) measurements on the samples at room temperature (Fig. 3a). Measured impedance spectra and corresponding fitting results are shown in Figure S1. For the material prepared through the solvent-based approach, the conductivity varied from 4.2×10^−6^ S cm^−1^ (175 °C) to 2.3×10^−6^ S cm^−1^ (250 °C). The maximum value of 1.7×10^−5^ S cm^−1^, similar to what has been reported in the original study^29^, was achieved at 200 °C. However, it seems that the conductivity is not strongly affected by the temperature. For instance, for the sample annealed at 175 °C, a relatively large fraction of unreacted LiI was still present, but the conductivity was comparable to that of the sample at 250 °C with mostly Li_4_PS_4_I. This result implies that other factors are at play too, likely associated with the solvent (DME) or its residues, which may have an effect on the grain boundaries.

Next, the conductivity of the materials prepared by solid-state synthesis, starting from the glassy phase 1.5Li_2_S–0.5P_2_S_5_–LiI (~ 1.2×10^−3^ S cm^−1^), was determined. In agreement with previous reports, annealing at 175 °C already led to a decrease to 6.6×10^−4^ S cm^−1^. This trend continued with increasing temperature, leading to conductivities of 1.5×10^−4^ and 6.4×10^−5^ S cm^−1^ at 200 and 250 °C, respectively. The overall decrease is due to the crystallization of Li_4_PS_4_I. Accordingly, structural disorder in the 1.5Li_2_S–0.5P_2_S_5_–LiI system seems beneficial to the ion-transport properties, in agreement with literature findings^25,32–34^.

The glassy precursor material was also used for the hot-press experiments. Here, by far the highest conductivities of 2.6×10^−3^ S cm^−1^ (175 °C) and 3.5×10^−3^ S cm^−1^ (200 °C) were achieved. A value of 7.3×10^−5^ S cm^−1^ was determined for the crystalline Li_4_PS_4_I (250 °C), which is virtually identical to that of the solid-state synthesis. The highest conductivities were found again for primarily amorphous samples, being more than twice that of the precursor. This is likely a result of the material’s densification.

The density of the pelletized samples used for the EIS measurements was calculated from their geometry (dimensions) and mass (Fig. 3b). For the solvent-based materials, it ranged from 1.47 g cm^−3^ at 175 °C to 1.53 g cm^−3^ at 250 °C. The maximum density of 2.38 g cm^−3^ was found for 200 °C, close to the theoretical crystallographic density of 2.45 g cm^−3^ for tetragonal Li_4_PS_4_I^29^. The change (increase/decrease) in sample density correlates well with the trend in conductivity from EIS. Hence, one can assume that the changes in conductivity for the samples prepared at 200 and 250 °C are rather due to mechanical (compression) effects, as the respective XRD patterns were virtually identical (Fig. 2a).

For the materials prepared by solid-state synthesis, an increase in density from 2.07 to 2.65 g cm^−3^ with increasing temperature from 175 to 250 °C was observed (2.58 g cm^−3^ at 200 °C). Apparently, progressive crystallization of Li_4_PS_4_I (Fig. 2b) led to the changes in density. It should be noted, nevertheless, that for both 200 and 250 °C, the crystallographic density was slightly exceeded. These deviations may stem from microstructural effects; yet, the presence of LiI (*ρ*_th_ = 4.09 g cm^−3^) and Li_6_PS_5_I (*ρ*_th_ = 2.29 g cm^−3^) impurities also affects the density.

For the hot-press samples, a high density of 4.57 g cm^−3^ was calculated for 175 °C. This is most likely due to compaction (consolidation) of the amorphous material (Fig. 2c). The density strongly decreased to 3.06 g cm^−3^ upon increasing the annealing temperature to 200 °C, which may be associated with the beginning of the nucleation of Li_4_PS_4_I. Although the respective XRD pattern changed quite significantly, the sample density marginally decreased further to 2.95 g cm^−3^ at 250 °C.

As expected, the density of the different samples varied with the synthesis temperature. However, establishing reliable correlations with the conductivity is difficult, as other parameters, such as the composition and microstructure (compressibility), exert some influence as well. The materials prepared by hot pressing revealed the largest densities among all samples, likely due to thermal softening and consolidation during synthesis. After annealing at 250 °C, similar room-temperature conductivities of 0.05–0.1 mS cm^−1^ were achieved for the solid-state and hot-press synthesis routes, while the solvent-based approach led to a material with an order of magnitude lower conductivity. In a broader context, (ignoring microstructural effects) these observations agree with the potentially negative effect that wet chemistry may have on the ionic conductivity, as also seen in the preparation of argyrodite SEs^35,36^.

**Figure 4:**
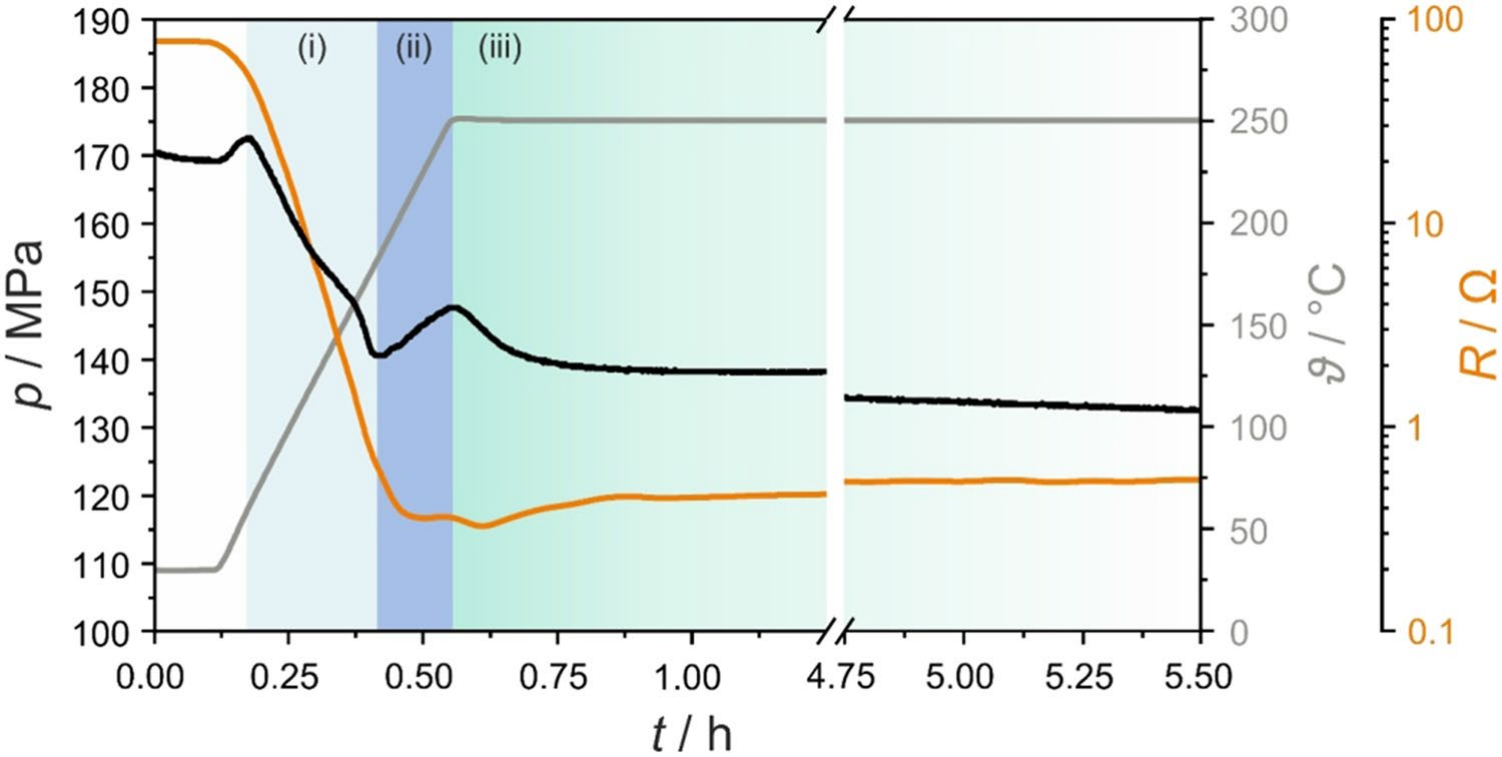
Synthesis *pTR*-diagram for Li_4_PS_4_I from glassy 1.5Li_2_S–0.5P_2_S_5_–LiI using a hot-press setup. Sample temperature, pressure and resistance versus the time are shown as gray, black and orange lines, respectively. Light blue, blue and green shaded areas indicate (i) precursor softening, (ii) nucleation of Li_4_PS_4_I and (iii) crystallization.

To gain more insights into the crystallization behavior of Li_4_PS_4_I from the glassy phase 1.5Li_2_S–0.5P_2_S_5_–LiI, a hot-press setup capable of monitoring temperature, pressure and electrical resistance simultaneously and in situ was utilized^31^. The initial pressure was set to 173 MPa (2 t) and after some equilibration period, the temperature was linearly increased to 250 °C at a heating rate of 500 °C h^−1^. As can be seen from the *pTR*-diagram in Fig. 4, the sample showed a resistance of 76 Ω (170 MPa, ~ 1.96 t) at room temperature, corresponding to 1.1 × 10^−3^ S cm^−1^. On subsequent temperature increase, both pressure and resistance decreased until ~ 175 °C. The effect of simultaneous pressure and resistance lowering can be attributed to thermally induced softening and the resulting consolidation of the sample, as also observed for 3.5Li_2_S–1.5P_2_S_5_ (Li_7_P_3_S_11_), for example^31^. In the course of heating to 250 °C, a linear pressure increase from 140 MPa (1.62 t) to 147 MPa (1.70 t) occurred and the resistance leveled off at ~ 0.36 Ω. Finally, an asymptotic pressure decrease was noticed during the dwell time at 250 °C, reaching 133 MPa (1.53 t) after 5.5 h, with the resistance slightly increasing and stabilizing at ~ 0.55 Ω. This fast increase and then slow decrease in pressure is indicative of the nucleation of Li_4_PS_4_I between ~ 175 and 250 °C and the onset of crystallization at 250 °C. This behavior agrees well the above XRD results (Fig. 2c). We note that *pTR*-diagrams for *ϑ* < 250 °C did not show such a pressure evolution (Figure S2).

Assuming constant nucleation and growth rates, the transformation at a given temperature follows sigmoidal kinetics, i.e., the product fraction increases exponentially with time at first and then asymptotically decreases. The Johnson–Mehl–Avrami–Kolmogorov (JMAK) model can describe this kind of behavior. In the present work, we tried to correlate the changes in pressure in the isothermal region at 250 °C (Fig. 4a) with the fraction of Li_4_PS_4_I (Figure S3a)^31^. Specifically, by applying the JMAK model to the pressure changes, a qualitative description of the crystallization kinetics can be derived. From the data, we calculated an Avrami exponent of 1.64(2), suggesting that the crystallization (crystal growth) occurs through a diffusion-controlled, non-ideal 1-dimensional/2-dimensional growth (Figure S3b)^37–39^.

**Figure 5:**
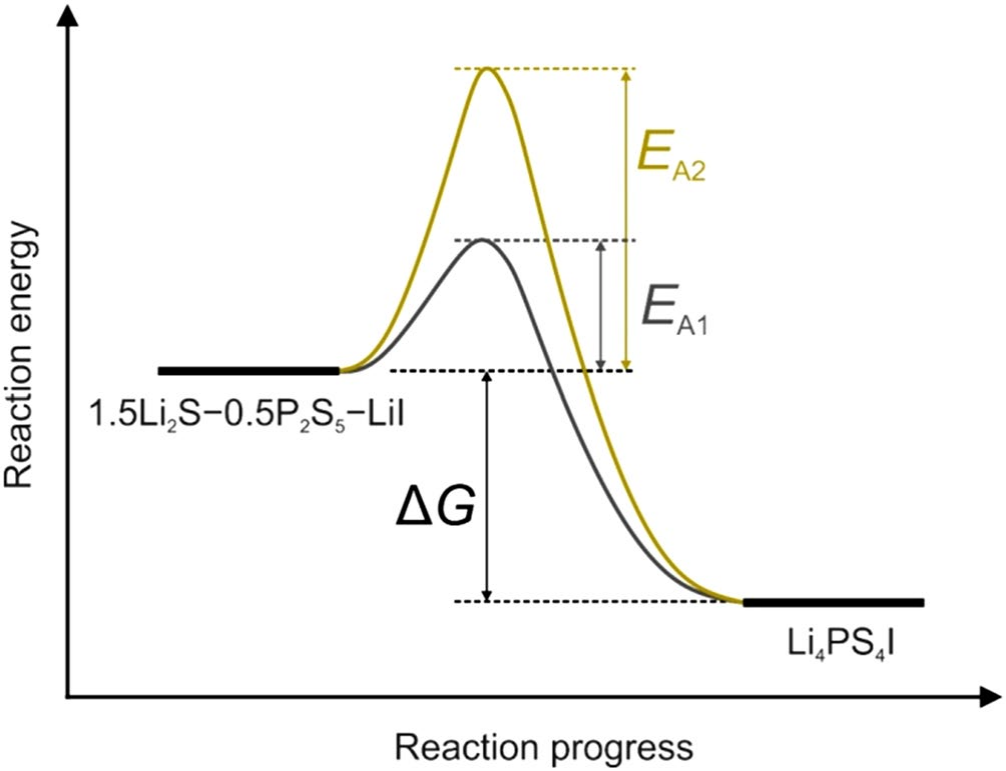
Reaction scheme for the preparation of Li_4_PS_4_I from glassy 1.5Li_2_S–0.5P_2_S_5_–LiI. In contrast to ambient pressure conditions with *E*_A1_, an increase in activation energy (nucleation barrier) *E*_A2_ is noticed when the synthesis is performed under pressure.

The crystallization temperature of 250 °C determined from the *pTR*-diagram was not consistent with differential scanning calorimetry (DSC) data recorded at a similar heating rate of 8.3 °C min^−1^ (Figure S4). The heat flow showed a sharp exothermic peak at 165 °C followed by a broad one centered at ~ 250 °C. Recently, we demonstrated by ex situ XRD that the former peak denotes the crystallization of Li_4_PS_4_I (*ϑ*_c_ ≈ 165 °C)^25^. Hence, we hypothesize that the large discrepancy is a result of the applied pressure during the hot-press experiment, increasing the activation energy for nucleation (Fig. 5) and delaying crystallization. Comparable observations of pressure affecting the phase-transformation kinetics have been made in the past for various inorganic materials^40,41^. In general, the atomic mobility is lowered as the pressure is increased. The rise in *ϑ*_c_ to ~ 250 °C may be due to the different densities (molar volumes) of the glassy 1.5Li_2_S–0.5P_2_S_5_–LiI and tetragonal Li_4_PS_4_I^42^. Assuming uniform nucleation during crystallization, the effect that pressure has on the nucleation activation energy can be expressed as $\left(\frac{\partial \Delta {G}^{\mathrm{*}}}{\partial p}\right)=-\frac{32\pi {\sigma }^{3}}{3}\frac{\Delta V}{{\left(\Delta G\right)}^{3}}$, where *σ* is the interfacial energy (not sensitive to pressure), Δ*G* = *G*_a_ – *G*_c_ is the difference in free energy between the amorphous and crystalline phases and Δ*V* = *V*_a_ – *V*_c_ represents the corresponding difference in molar volume^43,44^. For $\frac{\partial \left({\Delta G}^{*}\right)}{\partial p}>0$, pressure will increase the activation energy for nucleation. Because the crystallization of Li_4_PS_4_I is exothermic, see Figure S4 (with Δ*G* < 0), this implies a molar volume change of Δ*V* > 0. Although somewhat uncommon, the glassy 1.5Li_2_S–0.5P_2_S_5_–LiI may indeed be more dense, as the tetragonal Li_4_PS_4_I phase contains five crystallographic Li sites with an occupation of < 50% on average and therefore a large fraction of “free volume”. At the same time, however, we cannot fully rule out that the crystallization is kinetically hindered for the reason mentioned above.

**Figure 6:**
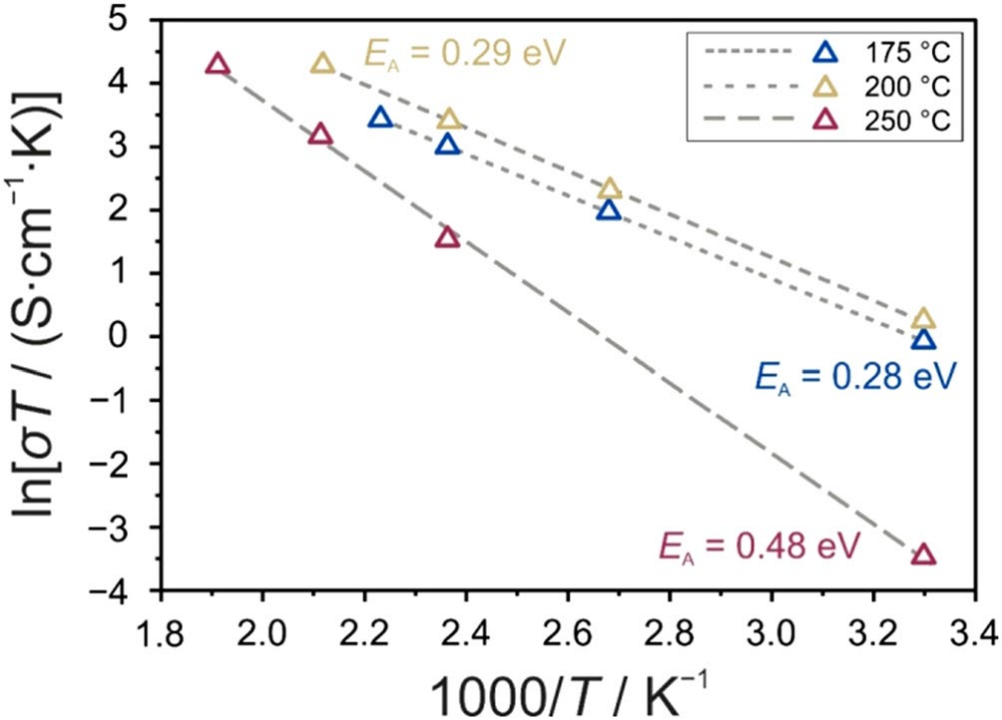
Arrhenius plots for the temperature dependence of conductivity for glassy 1.5Li_2_S–0.5P_2_S_5_–LiI heated at 175, 200 or 250 °C using a hot-press setup.

Finally, temperature-dependent EIS measurements were conducted on the glassy phase 1.5Li_2_S–0.5P_2_S_5_–LiI heated at the different target temperatures in the hot press (upon cooling to room temperature). As shown in Fig. 6, prior to the onset of crystallization of Li_4_PS_4_I, activation energies for conduction of 0.28 eV (175 °C) and 0.29 eV (200 °C) were calculated from the Arrhenius plots. These activation energies are similar to that of the pristine 1.5Li_2_S–0.5P_2_S_5_–LiI material (0.29 eV, Figure S5). However, the room-temperature conductivities were increased (2.6 × 10^−3^ and 3.5 × 10^−3^ S cm^−1^ vs 1.1 × 10^−3^ S cm^−1^). We hypothesize that two major effects play a role here. First, as mentioned before, annealing in the hot press causes sample densification, thereby minimizing porosity. Second, nanocrystalline LiI embedded in the amorphous thiophosphate matrix may provide facile (interfacial) conduction pathways (space-charge regions) for improved lithium diffusivity^45–48^. In contrast, a significantly higher activation energy of 0.48 eV and a much lower room-temperature conductivity of 7.3 × 10^−5^ S cm^−1^ were obtained for the sample crystallized at 250 °C.

## Discussion

In the present work, we have examined the preparation of Li_4_PS_4_I using three different routes, namely, wet-chemical, solid-state and hot-press synthesis. Structural changes upon annealing were probed by ex situ XRD, while the conductivity was measured by EIS. Using a specialized hot-press setup and DSC, we further provide real-time insights into the crystallization of Li_4_PS_4_I from glassy 1.5Li_2_S–0.5P_2_S_5_–LiI. Overall, the results confirm that (partially) amorphous solid electrolytes exhibit orders of magnitude higher ionic conductivity than the crystalline Li_4_PS_4_I phase (*P*4/*nmm*). Note that such solid electrolytes may be beneficial to the long-term SSB operation by alleviating the mechanical degradation^25,49^. When the samples are consolidated via hot pressing, significantly increased room-temperature conductivities ranging from 2.6 to 3.5 mS cm^–1^ can be achieved (unlike for the wet-chemical and solid-state synthesis routes). Such improvements in conductivity have also been reported for other thiophosphate-based Li-ion conductors^31,50,51^. In addition, we show that the crystallization temperature varies with the pressure applied during synthesis. Taken together, our findings emphasize the profound effects that the synthesis route, temperature and pressure have on the phase transformation (crystallization behavior) of quasi-ternary glasses in the *x*Li_2_S–*y*P_2_S_5_–*z*LiI system and their phase composition and charge-transport properties.

## Materials/methods

General. All manipulations were carried out under protective Ar atmosphere in a glovebox (MBraun; O_2_ < 0.1 ppm, H_2_O < 0.1 ppm) or using Schlenk-line techniques, if not stated otherwise. Li_2_S (Sigma Aldrich; 99.98%), P_2_S_5_ (Sigma Aldrich; 99%), LiI (Alfa Aesar; 99.99%) and anhydrous 1,2-dimethoxyethane (Sigma Aldrich; DME, 99.5%) were used as received.

Solvent-based synthesis. For the solvent-based synthesis of Li_4_PS_4_I, a two-step procedure was followed, as reported in the literature^29^. Specifically, 0.488 g Li_2_S (10.62 mmol) and 1.012 g P_2_S_5_ (4.553 mmol) were ground using a mortar and pestle and filled into a flask. Excess P_2_S_5_ was used to account for the loss during synthesis. Next, 40 mL DME was added under stirring and the dispersion kept stirring for 72 h. Subsequently, white precipitate was recovered through filtration, washed three times with 5 mL DME and dried in a vacuum overnight to yield Li_3_PS_4_∙DME. Next, 500 mg Li_3_PS_4_∙DME (1.851 mmol) was added to a solution of 247.7 mg LiI (1.851 mmol) in 10 mL DME and stirred for 24 h to form Li_3_PS_4_∙DME∙LiI. Excess solvent was removed in a vacuum. The recovered powder was first dried at 50 °C overnight and finally annealed at 175, 200 or 250 °C (~ 2 °C min^–1^ heating rate) under a dynamic vacuum for 12 h, followed by natural cooling.

Solid-state synthesis. Stoichiometric amounts of Li_2_S, P_2_S_5_ and LiI (1.5 g in total) were mixed in a planetary ball-mill (Fritsch), using a 70 mL zirconia jar with 20 zirconia balls of 10 mm diameter, for 1 h at 250 rpm and then for 15 h at 450 rpm, yielding glassy 1.5Li_2_S–0.5P_2_S_5_–LiI. For subsequent treatment, ~ 250 mg powder was pressed into 10 mm-diameter pellets and vacuum-sealed in quartz ampoules. Finally, the samples were annealed for 12 h at 175, 200 or 250 °C (5 °C min^–1^ heating rate) in a box furnace (Nabertherm), followed by natural cooling.

Hot-press synthesis. A detailed description and schematic drawings of the setup used are provided elsewhere^31^. All processing steps were done in an Ar glovebox. For each experiment, 300 mg glassy 1.5Li_2_S–0.5P_2_S_5_–LiI was loaded into the press die between two 12 mm-diameter Pt discs. Initially, the pressure was set to 173 MPa (2 t) and monitored further throughout the experiment. The sample was heated to the target temperature (175, 200 or 250 °C) at a rate of 500 °C h^−1^ and kept there for 5 h, followed by natural cooling.

X-ray diffraction (XRD). Powder XRD measurements were performed in borosilicate capillaries (Hilgenberg; 0.48 mm inner diameter, 0.01 mm wall thickness) using a STADI P diffractometer (STOE) equipped with a Cu-K_α1_ radiation source. Rietveld refinement analysis was performed as implemented in FullProf Suite (version July 2017)^52^. The Thompson-Cox-Hastings pseudo-Voigt function was used to describe the reflection shape. A fixed background was fitted to the data using a Chebyshev polynomial function with 24 terms. In the structural model, the unit-cell parameters, scale factor, zero shift and crystallite size broadening parameters were refined. Bond-valence energy landscapes (BVELs) were generated according to a method developed by Adams^53^, implemented in the program BondSTR of FullProf Suite. VESTA software (version 3.4.8) was used to generate crystal structure and BVEL images^54^.

Electrochemical impedance spectroscopy (EIS). EIS was measured from 0.1 Hz to 7 MHz, with a 20 mV voltage amplitude, using a SP-200 potentiostat (BioLogic). For the samples prepared by the wet-chemical and solid-state routes, an amount of 120 mg powder was compressed between two 10 mm-diameter stainless steel rods for 2 min at 440 MPa (3.5 t) using a specialized cell setup. A pressure of 63 MPa (0.5 t) was maintained during the measurement. For samples prepared by hot pressing, EIS was directly performed in the setup (after annealing and subsequent cooling to room temperature). To calculate the conductivity and sample density, the pellet thickness was determined afterwards. Impedance spectra were analyzed with the EC-Lab software (BioLogic) assuming a *R*_1_(*R*_2_*Q*_2_)*Q*_3_ equivalent circuit in case of the solvent-based and solid-state synthesis routes. For the other samples, *R* was determined from the intercept of a linear fit of the date with the *x*-axis.

Differential scanning calorimetry (DSC). DSC measurements were conducted on a DSC 204 F1 Phoenix (NETZSCH). The samples were sealed in Al crucibles and measurements were done under protective Ar flow at a heating rate of 5 or 8.3 °C min^−1^ from 30 to 400 °C.

## Supplementary Information

Supplementary Information.

## Data Availability

The datasets generated during and/or analyzed during the current study are available from the corresponding author on reasonable request.
