# Supplementary material for: Influence of synthesis parameters on crystallization behavior and ionic conductivity of the Li4PS4I solid electrolyte
Source: Sci Rep. 2021 Jul 7;11:14073. doi: 10.1038/s41598-021-93539-4 (PMC8263741; doi:10.1038/s41598-021-93539-4)
Supplement: Supplementary file 1 — Supplementary Information. [file 41598_2021_93539_MOESM1_ESM.docx]

Supporting Information

Influence of Synthesis Parameters on Crystallization Behavior and Ionic Conductivity of the Li_4_PS_4_I Solid Electrolyte

Florian Strauss,^1,^* Jing Lin,^1^ Jürgen Janek,^1,2^ and Torsten Brezesinski^1^

^1^Battery and Electrochemistry Laboratory, Institute of Nanotechnology, Karlsruhe Institute of Technology (KIT), Hermann-von-Helmholtz-Platz 1, 76344 Eggenstein-Leopoldshafen, Germany.

^2^Institute of Physical Chemistry & Center for Materials Science, Justus-Liebig-University Giessen, Heinrich-Buff-Ring 17, 35392 Giessen, Germany.

*Email: [florian.strauss@kit.edu](mailto:florian.strauss@kit.edu)


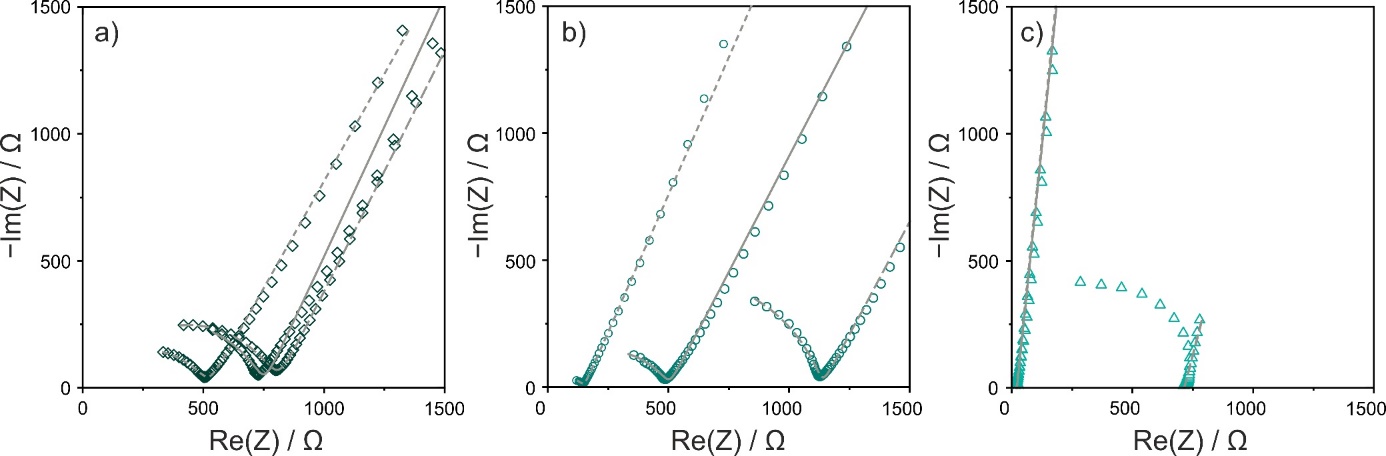


**Figure S1.** Room-temperature impedance spectra and corresponding fitting results for Li_4_PS_4_I prepared by (**a**) wet-chemical, (**b**) solid-state or (**c**) hot-press synthesis at different temperatures of 175 (short-dashed lines), 200 (solid lines) and 250 °C (long-dashed lines). Note that the pellet dimensions varied among the samples. Calculated conductivities are shown in Figure 3a in the manuscript.


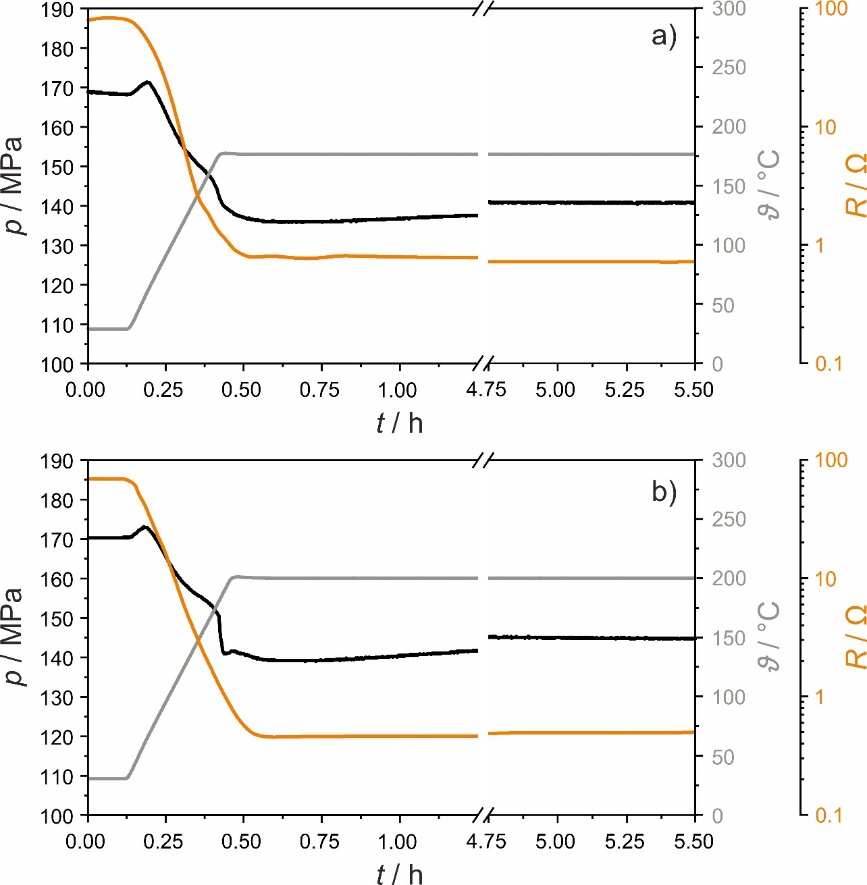


**Figure S2.** Synthesis *pTR*-diagrams for 1.5Li_2_S-0.5P_2_S_5_-LiI heated to (**a**) 175 and (**b**) 200 °C in the hot press. Sample temperature, pressure and resistance versus the time are shown as gray, black and orange lines, respectively.


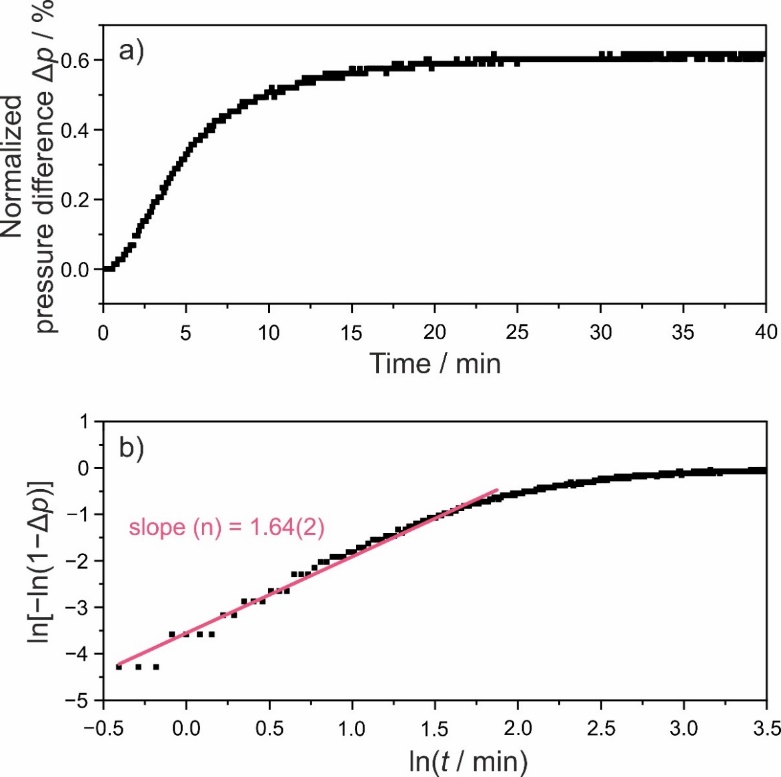


**Figure S3.** (**a**) Normalized pressure difference versus the time showing sigmoidal curve shape (see ref.^1^ for details on the calculation). *t* = 0 was set at the beginning of the isothermal region at 250 °C, as shown in Figure 4 in the manuscript. (**b**) Corresponding plot of ln[–ln(1–Δ*p*)] versus ln(*t*/min) for the onset of crystallization of Li_4_PS_4_I and linear curve fitting.


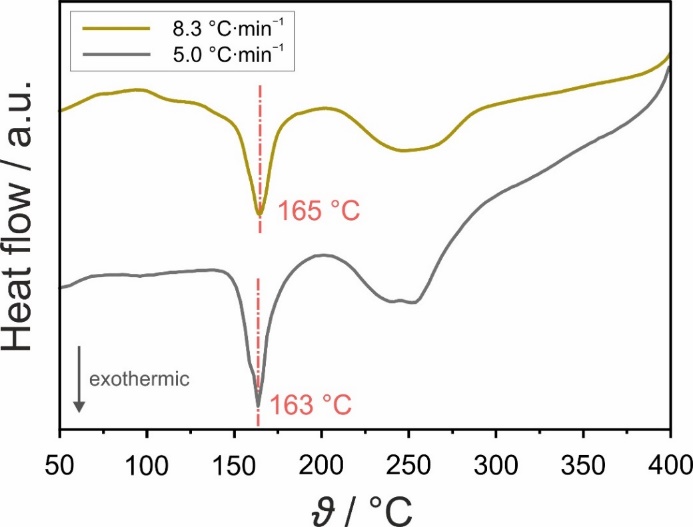


**Figure S4.** DSC curve for 1.5Li_2_S-0.5P_2_S_5_-LiI heated to 400 °C at two different rates.


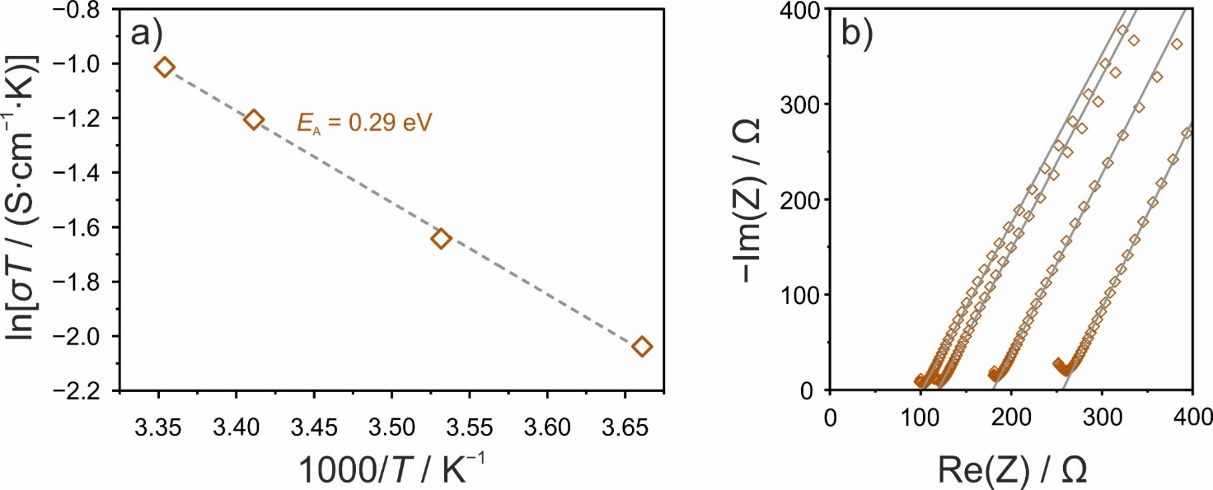


**Figure S5.** (**a**) Arrhenius plot for the temperature dependence of the conductivity for pristine 1.5Li_2_S-0.5P_2_S_5_-LiI and (**b**) corresponding impedance spectra and fitting results (gray lines).

**Table S1.** Reliability factors for the Rietveld refinement of Li_4_PS_4_I (see Figure 2d-f in the manuscript) prepared at 250 °C by wet-chemical, solid-state or hot-press synthesis.

| **Reliability factors** | **Wet-chemical** | **Solid-state** | **Hot-press** |
| --- | --- | --- | --- |
| *R*_wp_ / % | 16.2 | 26.6 | 22.9 |
| *χ*^2^ | 4.28 | 11.7 | 4.44 |

References

1. Busche, M. R. *et al*. In situ monitoring of fast Li-ion conductor Li_7_P_3_S_11_ crystallization inside a hot-press setup. *Chem. Mater.* **28**, 6152-6165 (2016).
